# Safety with Ocrelizumab in Rheumatoid Arthritis: Results from the Ocrelizumab Phase III Program

**DOI:** 10.1371/journal.pone.0087379

**Published:** 2014-02-03

**Authors:** Paul Emery, William Rigby, Paul P. Tak, Thomas Dörner, Ewa Olech, Carmen Martin, Laurie Millar, Helen Travers, Elena Fisheleva

**Affiliations:** 1 Leeds Institute of Rheumatic and Musculoskeletal Medicine, University of Leeds, Leeds, United Kingdom; 2 NIHR Leeds Musculoskeletal Biomedical Research Unit, Leeds Teaching Hospitals NHS Trust, Leeds, United Kingdom; 3 The Geisel School of Medicine at Darthmouth, Lebanon, New Hampshire, United States of America; 4 Academic Medical Centre, University of Amsterdam, Amsterdam, the Netherlands; 5 Charité University Hospitals and DRFZ, Berlin, Germany; 6 University of Nevada School of Medicine, Las Vegas, Nevada, United States of America; 7 Roche Products, Ltd, Welwyn Garden City, United Kingdom; CUNY, United States of America

## Abstract

**Objective:**

The objective was to determine the safety of ocrelizumab (OCR) in patients with rheumatoid arthritis (RA).

**Methods:**

This was an analysis of the double-blind, placebo-controlled periods and long-term follow-up of 4 OCR phase III trials in RA (SCRIPT, STAGE, FILM and FEATURE). Safety data per study and the results of a meta-analysis of serious infectious events (SIEs) are presented.

**Results:**

Overall, 868 patients received placebo, 1064 patients OCR 200 mg×2 (or 400 mg×1) (OCR200), and 827 patients OCR 500 mg×2 (OCR500) plus background methotrexate (MTX) at baseline and 24 weeks. During the double-blind, placebo-controlled periods, the incidence of adverse events and serious adverse events was comparable between the OCR+MTX and placebo +MTX groups. Infusion-related reactions were more common with OCR+MTX and decreased in frequency with subsequent infusions. Serious infusion-related reactions were rare (0.1%). Serious infections occurred more frequently with OCR500+MTX. In the meta-analysis, a statistically significant difference from placebo +MTX in incidence of SIEs per 100 patient-years of 2.4 (95% CI, 0.3–4.5) was observed with OCR500+MTX, but not with OCR200+MTX (0.6; 95% CI, −1.3 to 2.4). Patients recruited in Asia exhibited a higher risk of serious infections (hazard ratio, 1.78; 95% CI, 1.03–3.06). The incidence of human anti-human antibodies was <5%. Long-term follow-up indicated no differences in malignancy rates between the treatment groups. There was no apparent difference in time to B-cell repletion between the OCR dose groups.

**Conclusions:**

In placebo-controlled clinical trials of RA, OCR500+MTX was associated with a higher risk of serious infections compared with placebo +MTX. The safety profile of OCR 200+MTX was comparable with placebo+MTX.

**Trial Registration:**

STAGE Clinical Trials.gov NCT00406419

SCRIPT Clinical Trials.gov NCT00476996

FILM Clinical Trials.gov NCT00485589

FEATURE Clinical Trials.gov NCT00673920

## Introduction

Although the immunopathogenesis of rheumatoid arthritis (RA) is not fully understood, accumulating evidence suggests that B cells have multiple potential roles through both antibody-dependent and antibody-independent pathways [1], [2]. Rituximab is a chimeric mouse-human monoclonal antibody that depletes CD20+ B cells and has been shown to be an effective therapy in patients with RA [3]–[7]. Pooled analysis of long-term safety data from patients receiving rituximab within a global clinical trial program indicated that rituximab is well tolerated over time and during multiple courses of treatment [8], [9]. However, as with all chimeric antibodies, immunogenicity may be a potential concern. A safety analysis showed that 11% of patients with RA developed a titer positive for human anti-chimeric antibody (HACA) on at least one occasion during treatment with rituximab [8]. The presence of HACAs was not associated with the development of infusion-related reactions (IRRs) or loss of efficacy on retreatment. Thus, the clinical impact of HACA directed at rituximab remains unclear.

Ocrelizumab (rhuMAb 2H7, [OCR]) is a humanized anti-CD20 monoclonal antibody. In vitro characterization of OCR demonstrated enhanced antibody-dependent cell-mediated cytotoxicity and reduced complement-dependent cytotoxicity compared with rituximab (unpublished data), although the clinical implications of these differences remain unclear. The efficacy and safety of OCR in RA has been evaluated in a robust phase III clinical trial program in a broad spectrum of patients [10]–[13]. In May 2010, OCR development in RA was terminated as a result of the overall risk-benefit assessment from the 2 pivotal phase III studies STAGE and SCRIPT. The efficacy and safety profiles of the OCR 200 mg (OCR200) and OCR 500 mg (OCR500) dosing regimens led the sponsors to conclude that OCR did not demonstrate an additional benefit over existing therapies, including rituximab for patients with RA, and that an application for regulatory approval of OCR in RA was not warranted. This paper presents the key safety outcomes of the 4 phase III OCR trials in RA to provide an overview of the safety of OCR in patients with RA and background methotrexate (MTX) treatment.

## Patients and Methods

The CONSORT checklist is available as supporting information; see Checklist S1.

### Ethics Statement

These studies were conducted at 686 sites across more than 20 different countries in accordance with the ethical principles of the Declaration of Helsinki. Ethical approval from the local institutional review board at each study center was obtained before the start of each study and all patients provided written informed consent. All studies included were previously registered with ClinicalTrials.gov (registration nos. NCT00406419, NCT00476996, NCT00485589 and NCT00673920).

### Patients

Patients included in the analyses were participants in 1 of 4 OCR phase III trials [10]–[13]. The analysis population represented a broad spectrum of patients, ranging from patients with early RA who were MTX-naive (FILM [12], registration no. NCT00485589) to patients with advanced RA disease who were refractory to disease-modifying antirheumatic drugs (DMARDs) (FEATURE [13], registration no. NCT00673920 and STAGE [10], registration no. NCT00406419) and/or anti-TNFs (SCRIPT [11], registration no. NCT00476996). The overwhelming majority of patients received background MTX; leflunomide could also be used instead of MTX in SCRIPT.

### Study Designs

All 4 trials were phase III international, randomized, and double-blind, placebo-controlled (DBPC); STAGE was conducted at 209 centers in 24 countries, SCRIPT was conducted at 227 centers in 25 countries, FEATURE was conducted at 96 centers in 14 countries and FILM was conducted at 154 centers in 21 countries. The study designs and numbers of patients randomized were reported previously [10]–[13] and are summarized in Table 1. During the DBPC period of STAGE, SCRIPT and FILM, patients received treatment on Days 1 and 15 followed by a retreatment course at Weeks 24 and 26 (patients in FILM were eligible for 2 additional retreatment courses at Weeks 52 and 54, and Weeks 76 and 78). At the end of the DBPC period in FEATURE (Week 24), all patients were re-randomized to receive either OCR200 ×2+MTX or OCR 400 mg (OCR400) +MTX for a 24-week double-blind (but not placebo-controlled) treatment period. After completion of the double-blind period (48 weeks [SCRIPT, STAGE and FEATURE] and 104 weeks [FILM]), patients entered an open-label extension, where they were treated with OCR500 ×2+MTX (SCRIPT, STAGE and FILM) or OCR400+MTX (FEATURE) at the discretion of the investigator. At the time that FILM was terminated, all patients had completed 52 weeks of DBPC treatment and only a few had completed 104 weeks and entered the open-label extension. Therefore, analysis of the DBPC period for FILM included only the Week 52 data. At the time that FEATURE, SCRIPT and STAGE were terminated, all patients had completed the double-blind 48-week period. Upon withdrawal from treatment, all patients were required to continue in safety follow-up (SFU) for at least 48 weeks from the first infusion of their last course and until their CD19+ B-cell counts either returned to baseline level or the lower limit of normal (80 cells/µl), whichever was lower.

**Table 1:** Summary of 4 Phase III Studies of Ocrelizumab.

Trial Name	Patients Treated, n	RA Characteristics	Treatment Groups+MTX^a^	Duration of PBO-Controlled Period, weeks
**STAGE**	1006	MTX-IR; 51% to 54% steroid use; Baseline DAS28 ≈6.4	PBO (n = 320); OCR200 (n = 343); OCR500 (n = 343)	48
**SCRIPT**	836	TNF-IR; 56% to 62% steroid use; Baseline DAS28 ≈6.5	PBO (n = 277); OCR200 (n = 277); OCR500 (n = 282)	48
**FEATURE**	312	MTX-IR/biological DMARD-IR; 52% to 59% steroid use; Baseline DAS28 ≈6.5	PBO (n = 64); OCR200 (n = 131); OCR400 (n = 117)	24
**FILM**	605	MTX-naïve; 39% to 42% steroid use; Baseline DAS28 ≈7.0	PBO (n = 207); OCR200 (n = 196); OCR500 (n = 202)	104^b^

Abbreviations: DAS28, disease activity score in 28 joints; DMARD, disease-modifying antirheumatic drug; IR, inadequate responder; MTX, methotrexate; OCR200, ocrelizumab 200 mg×2; OCR500, ocrelizumab 500 mg×2; PBO, placebo; RA, rheumatoid arthritis; TNF, tumor necrosis factor.

a All patients in all studies received background MTX 7.5 to 25 mg/week (7.5–20 mg/week in FILM), except for in SCRIPT, in which MTX or leflunomide was permitted. Treatment with corticosteroids (≤10 mg/day prednisolone or equivalent) was permitted in all studies provided the dose was stable 4 weeks prior to baseline.

b Study terminated early. Safety evaluation conducted for 52-week data.

### Safety Assessments

In each trial, clinical adverse events (AEs) and serious AEs (SAEs) were recorded, and the intensity of AEs was graded using the National Cancer Institute Common Toxicity Criteria (version 3) and coded according to MedDRA (version 12.1). Malignancies were identified using the wide standard MedDRA query Malignant or Unspecified Tumors. Serious infectious events (SIEs) also included those requiring intravenous antibiotics. IRRs and symptoms were recorded on a specifically designed page of the case report form.

### Immunogenicity and Pharmacodynamics

The primary pharmacodynamic (PD) marker for OCR is the presence of CD20+ B cells in the blood. Because the presence of OCR in serum could confound assays of CD20+ cells, CD19 was used to measure the levels of peripheral B cells following treatment (limit of detection, 0.05×10^9^ cells/l in conventional flow cytometry). In each trial, serum samples were collected at prespecified time points for the determination of human anti-human antibodies (HAHAs) and B-cell levels (CD19+ cells). A bridging format enzyme-linked immunosorbent assay was used to determine HAHA titers. All positive samples were further confirmed by competitive binding to anti-IgM, followed by implementation of an additional decision tree to confirm or reject true positivity.

### Statistical Analysis

Safety and PD analyses were conducted on the safety population, which included all patients in each trial who were randomized, received any part of an infusion of study drug, and underwent at least one assessment of safety. Evaluation of the safety data for each study led to the conduct of a fixed-effects meta-analysis of SIEs. The incidence rate difference in SIEs from placebo (PBO)+MTX during the DBPC period, weighted by study size was calculated for both dose groups using data from all four studies (STAGE, SCRIPT, FEATURE and FILM). An exploratory, hypothesis-generating analysis of risk factors for SIEs was performed on STAGE, SCRIPT and FILM DBPC pooled data sets. The multivariate approach (COX regression models) investigated treatment group as a risk factor, with baseline covariates that included but were not limited to age, body mass index, body surface area, weight, race, region, previous use of biological and nonbiological DMARDs, MTX dose, corticosteroid use, RA disease duration, presence of selected comorbid conditions and study.

All available malignancy data from baseline to long-term SFU (up to 5 years of follow-up) in the 4 trials were pooled. Immunogenicity results included all data available for the DBPC periods.

PD data were analyzed using Kaplan-Meier methodology and included all data available after each patient completed at least 72 weeks of SFU after the last dose in each study.

In all analyses in which the FEATURE study was included, patients who received OCR200 or OCR400+MTX were grouped together in the OCR200+MTX group.

## Results

### Patient Population

**Figure 1:**
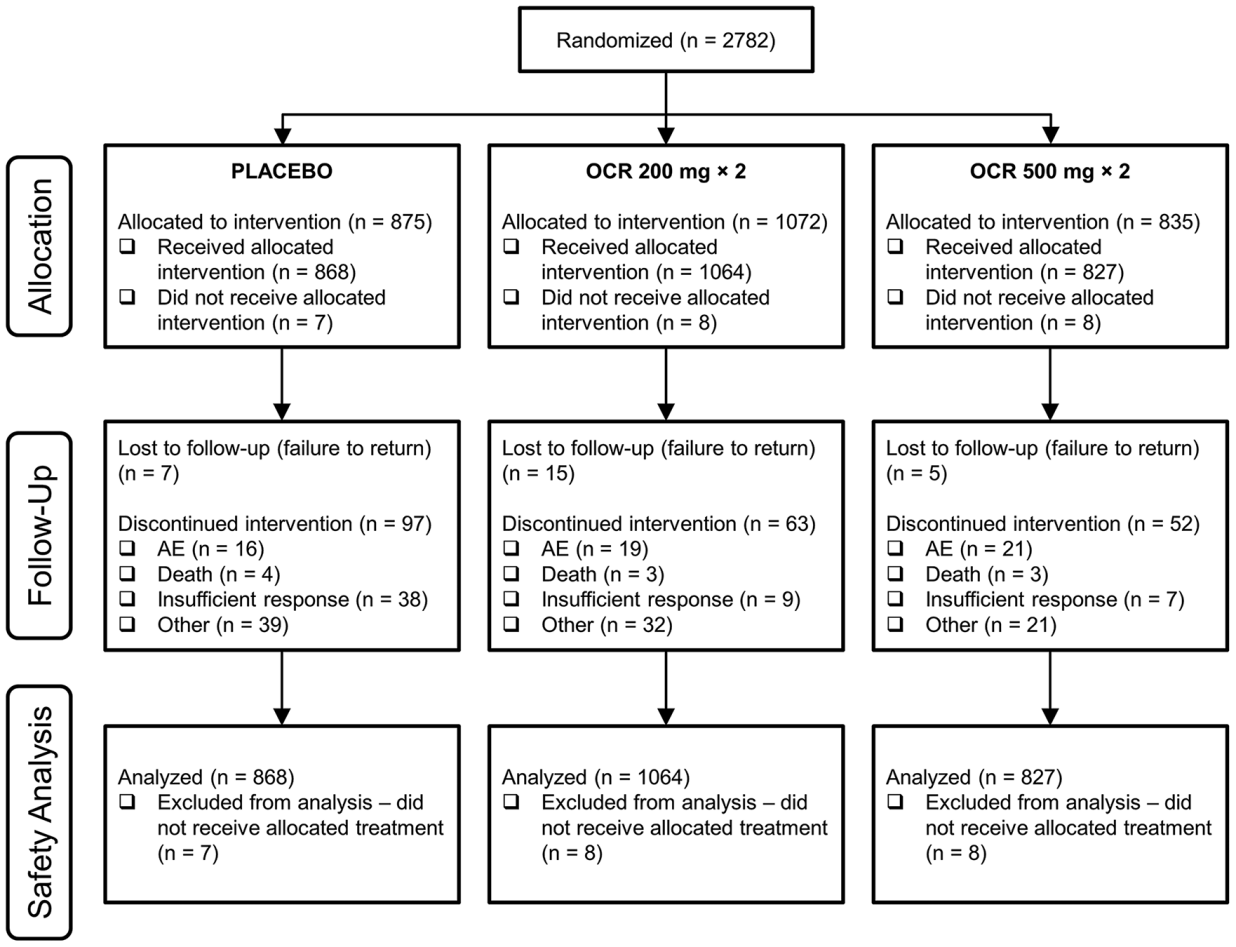
Patient disposition flow diagram of pooled safety population.

The safety analysis population comprised 2759 patients (868 patients treated with PBO+MTX, 1064 with OCR200+MTX and 827 with OCR500+MTX; Figure 1). The majority of patients were female and white and had a mean age ranging from approximately 49 to 55 years (Table 2). Disease duration varied because of the different patient populations. Patients in SCRIPT had long-standing RA, with a duration of approximately 11 to 12 years; patients in FILM had a considerably shorter disease duration of approximately 1.2 years. Corticosteroid (<10 mg/d) use varied from 39% to 42% in FILM to 56% to 62% in SCRIPT. In SCRIPT, leflunomide was received by 10.1%, 15.2% and 14.5% of the PBO+MTX, OCR200+MTX and OCR500+MTX groups, respectively, with mean doses of 19.6, 18.3 and 17.4 mg/d, respectively. All other patients in SCRIPT and all patients in the other trials received concomitant MTX.

**Table 2:** Baseline Patient Characteristics in Pooled Safety Populations^a^.

Characteristic	PBO+MTX^b^	OCR200+MTX^b^	OCR500+MTX^b^
	(n = 868)	(n = 1064)	(n = 827)
Female, %	74.0 to 87.5	77.3 to 82.5	80.0 to 83.7
White, %	68.8 to 74.4	65.9 to 73.0	67.0 to 75.6
Mean age, years	49.2 to 54.2	50.8 to 54.5	48.6 to 53.8
Mean RA disease duration, years	1.2 to 11.8	1.2 to 12.7	1.2 to 12.3
Serological status, %			
— RF+/ACPA+	83.0 to 87.9	80.2 to 87.8	77.1 to 86.1
— RF+/ACPA−	4.8 to 8.5	6.6 to 9.7	4.5 to 8.5
— RF–/ACPA+	6.3 to 9.4	5.1 to 11.2	to 15.3
— RF–/ACPA−	0 to 1.6	0 to 1.2	0.7 to 1.5
SJC (66 joints), mean	16.6 to 21.1	16.5 to 19.4	17.1 to 19.5
TJC (68 joints), mean	26.0 to 31.6	26.2 to 30.8	26.4 to 30.0
CRP (mg/dl), mean	2.4 to 3.8	1.8 to 3.5	1.9 to 3.4
ESR (mm/h), mean	46.7 to 60.0	44.5 to 55.8	45.5 to 58.1
HAQ-DI, mean	1.5 to 1.8	1.5 to 1.8	1.5 to 1.7
DAS28-ESR, mean	6.4 to 7.0	6.4 to 7.0	6.4 to 6.9
Oral corticosteroid use, %	40 to 62	39 to 58	42 to 56

Abbreviations: ACPA, anti-citrullinated peptide antibody; CRP, C-reactive protein; DAS28, disease activity score in 28 joints; ESR, erythrocyte sedimentation rate; HAQ-DI, Health Assessment Questionnaire Disease Index; MTX, methotrexate; OCR200, ocrelizumab 200 mg×2; OCR500, ocrelizumab 500 mg×2; PBO, placebo; RA, rheumatoid arthritis; RF, rheumatoid factor; SJC, swollen joint count; TJC, tender joint count.

a Data shown as ranges (minimum and maximum values) across the 4 trials.

b All patients in all studies received background MTX 7.5 to 25 mg/week (7.5 to 20 mg/week in FILM), except for in SCRIPT, in which MTX or leflunomide was permitted.

### Overall Safety Profile

In all 4 trials, the incidence of all AEs during the DBPC periods was comparable in the PBO+MTX–treated and OCR+MTX–treated patients (Table 3). Grade 3 AEs were relatively infrequent, occurring in approximately 5% to 10% of patients across the treatment groups, with no clear differences between the PBO+MTX and OCR+MTX groups. The incidence of grade 4 AEs was 0% to 2.5%. AEs leading to patient withdrawal were infrequent; the most common in all 4 trials were IRRs and infections. Patients who received OCR500+MTX in FILM had a higher incidence of AEs leading to withdrawal than did patients who received PBO+MTX (5.9% vs 1.0%). Although the incidence of SAEs varied across the trials, there were no clear differences in general between the PBO+MTX and OCR+MTX groups or between the different dose groups (Table 3); the percentages of patients reporting ≥1 SAE were approximately 8% to 14% (OCR200+MTX) and 11% to 14% (OCR500+MTX), compared with 8% to 12% (PBO+MTX). The most common SAEs overall were infections and infestations. In STAGE and FEATURE, the occurrence of SAEs in other system organ classes was infrequent and comparable across treatment groups. In SCRIPT, serious musculoskeletal and connective tissue disorders were reported more frequently by patients in the PBO+MTX group (4.3%) compared with the OCR200+MTX (2.5%) and OCR500+MTX (2.5%) groups; this difference was mainly driven by an increased reporting of “exacerbation of RA.” The occurrence of SAEs in other system organ classes in SCRIPT was infrequent and comparable across treatment groups. In FILM, SAEs classified as respiratory, thoracic, and mediastinal disorders occurred more frequently with OCR500+MTX (2.5%) than with OCR200+MTX (0.5%) and PBO+MTX (0%); the most common SAE in this body system was interstitial lung disease, which was reported in 3 patients in the OCR500+MTX group. The occurrence of other body-system SAEs was infrequent and comparable across treatment groups.

**Table 3:** Summary of Safety During the Double-Blind, Placebo-Controlled Periods^a^.

Safety Profile	PBO+MTX^b^	OCR200+MTX^b^	OCR500+MTX^b^
**STAGE (MTX-IR)**			
Patients, n	320	343	343
Any AE, n (%)	254 (79.4)	282 (82.2)	287 (83.7)
— Grade 3, n (%)	25 (7.8)	20 (5.8)	25 (7.3)
— Grade 4, n (%)	2 (<1)	2 (<1)	2 (<1)
— Serious, n (%)	37 (11.6)	26 (7.6)	38 (11.1)
AEs leading to withdrawal, n (%)	5 (1.6)	5 (1.5)	6 (1.7)
Deaths, n (%)	1 (<1)	0 (0.0)	3 (<1)
IRRs, n (%)	31 (9.7)	69 (20.1)	80 (23.3)
— Serious, n (%)	0 (0.0)	1 (<1)	1 (<1)
Infections, n (%)	173 (54.1)	188 (54.8)	194 (56.6)
— Serious, n (%)	10 (3.1)	11 (3.2)	21 (6.1)
Malignancies, n (%)	6 (1.9)	3 (<1)	4 (1.2)
**SCRIPT (TNF-IR)**			
Patients, n	277	277	282
Any AE, n (%)	227 (81.9)	232 (83.8)	238 (84.4)
— Grade 3, n (%)	28 (10.1)	25 (9.0)	28 (9.9)
— Grade 4, n (%)	1 (<1)	2 (<1)	3 (1.1)
— Serious, n (%)	32 (11.6)	40 (14.4)	32 (11.3)
AEs leading to withdrawal, n (%)	10 (3.6)	11 (4.0)	7 (2.5)
Deaths, n (%)	1 (<1)	0 (0.0)	0 (0.0)
IRRs, n (%)	30 (10.8)	53 (19.1)	67 (23.8)
— Serious, n (%)	0 (0.0)	0 (0.0)	0 (0.0)
Infections, n (%)	143 (51.6)	150 (54.2)	164 (58.2)
— Serious, n (%)	7 (2.5)	14 (5.1)	12 (4.3)
Malignancies, n (%)	5 (1.8)	7 (2.5)	2 (<1)
**FEATURE (MTX-IR/biological DMARD-IR)**			
Patients, n	64	248	N/A
Any AE, n (%)	40 (62.5)	162 (65.3)	—
— Grade 3, n (%)	4 (6.3)	8 (3.2)	—
— Grade 4, n (%)	0 (0.0)	0 (0.0)	—
— Serious, n (%)	5 (7.8)	5 (2.0)	—
AEs leading to withdrawal, n (%)	2 (3.1)	3 (1.2)	—
Deaths, n (%)	0 (0.0)	0 (0.0)	—
IRRs, n (%)	7 (10.9)	53 (21.4)	—
— Serious, n (%)	0 (0.0)	0 (0.0)	—
Infections, n (%)	24 (37.5)	90 (36.3)	—
— Serious, n (%)	2 (3.1)	5 (2.0)	—
Malignancies, n (%)	0 (0.0)	1 (<1)	—
**FILM (MTX-naïve)**			
Patients, n	207	196	202
Any AE, n (%)	167 (80.7)	171 (87.2)	167 (82.7)
— Grade 3, n (%)	16 (7.7)	18 (9.2)	24 (11.9)
— Grade 4, n (%)	0 (0.0)	1 (<1)	5 (2.5)
— Serious, n (%)	21 (10.1)	18 (9.2)	28 (13.9)
AEs leading to withdrawal, n (%)	2 (1.0)	3 (1.5)	12 (5.9)
Deaths, n (%)	2 (1.0)	2 (1.0)	1 (<1)
IRRs, n (%)	18 (8.7)	52 (26.5)	54 (26.7)
— Serious, n (%)	0 (0.0)	0 (0.0)	1 (<1)
Infections, n (%)	106 (51.2)	101 (51.5)	105 (52.0)
— Serious, n (%)	6 (2.9)	5 (2.6)	10 (5.0)
Malignancies, n (%)	2 (1.0)	0 (0.0)	1 (<1)

Abbreviations: AE, adverse event; DMARD, disease-modifying antirheumatic drug; IR, inadequate responder; IRR, infusion-related reaction; MTX, methotrexate; OCR200, ocrelizumab 200 mg×2; OCR500, ocrelizumab 500 mg×2; PBO, placebo; TNF, tumor necrosis factor.

a Multiple events in individual patients were only counted once. Serious infections were defined as those requiring intravenous antibiotics and/or hospitalization or classified as serious by the investigator. Any opportunistic infection was classified as a serious infection.

b All patients in all studies received background MTX 7.5 to 25 mg/week (7.5 to 20 mg/week in FILM), except for in SCRIPT, in which MTX or leflunomide was permitted.

### Infusion-Related Reactions

The most common AEs overall were IRRs. The incidence of IRRs was approximately 2 to 3 times higher in the OCR+MTX group relative to the PBO+MTX group (Table 3). The highest incidence of IRRs occurred during and following the first infusion of the first course; the second infusion was tolerated better, and IRRs became less frequent with subsequent infusions. The most common symptoms were pruritus, pyrexia, flushing, laryngeal/throat irritation, headache, nausea, rash, chills/rigors, hypertension, urticaria and dizziness. IRRs were reported slightly more frequently with OCR500+MTX than with OCR200+MTX in both STAGE and SCRIPT but at a similar frequency with both OCR+MTX doses in FILM. Only 2 patients in STAGE and 1 patient in FILM reported a serious IRR. The 2 serious IRRs that occurred in STAGE were recorded for 1 patient in each of the 2 OCR+MTX groups. Both occurred during the first infusion of the first course and resolved following symptomatic treatment. In addition, 1 patient (OCR500+MTX) had an anaphylactoid reaction that began 45 min after the start of the first infusion of the first course. The reaction resolved without sequelae following symptomatic treatment. One patient in the OCR500+MTX group of FILM reported a serious IRR, which occurred approximately 12 hours after the second infusion of the second course. This patient experienced weakness, headache, elevated blood pressure and increased heart rate. Following hospitalization, the patient received antihypertensive medication and the elevated blood pressure resolved within 24 hours; the results of an electrocardiogram were normal.

### Human Anti-Human Antibodies

At baseline, pre-infusion, 0.6% of all patients were HAHA-positive—a result that was expected given the specificity and sensitivity of the assay used. In all 4 trials, the proportion of patients who developed HAHAs during the DBPC periods was low and comparable between the OCR+MTX and PBO+MTX groups. In FILM, the incidence of HAHAs over 52 weeks was 4/202 (2.0%), 2/194 (1.0%), and 8/201 (4.0%) in the PBO+MTX, OCR200+MTX and OCR500+MTX groups, respectively. The corresponding HAHA incidences in SCRIPT over 48 weeks were 5/274 (1.8%), 8/273 (2.9%) and 7/277 (2.5%), respectively, and in STAGE were 9/318 (2.8%), 16/338 (4.7%) and 7/339 (2.1%), respectively. In FEATURE, the HAHA incidence ranged from 0% in patients who received PBO followed by OCR 200 mg×2 to 10.7% (3/28) in patients who received PBO followed by OCR 400 mg×1. Among all patients who were HAHA-positive post-baseline, there was no apparent association between HAHA positivity and corresponding CD19 counts or DAS28 scores in any of the treatment groups across the 4 trials. Of the 3 patients who experienced a serious IRR, none was HAHA-positive at any time point tested; of the patients who were HAHA-positive, 4 experienced IRRs—all grade 1 or 2.

### Serious Infections

**Figure 2:**
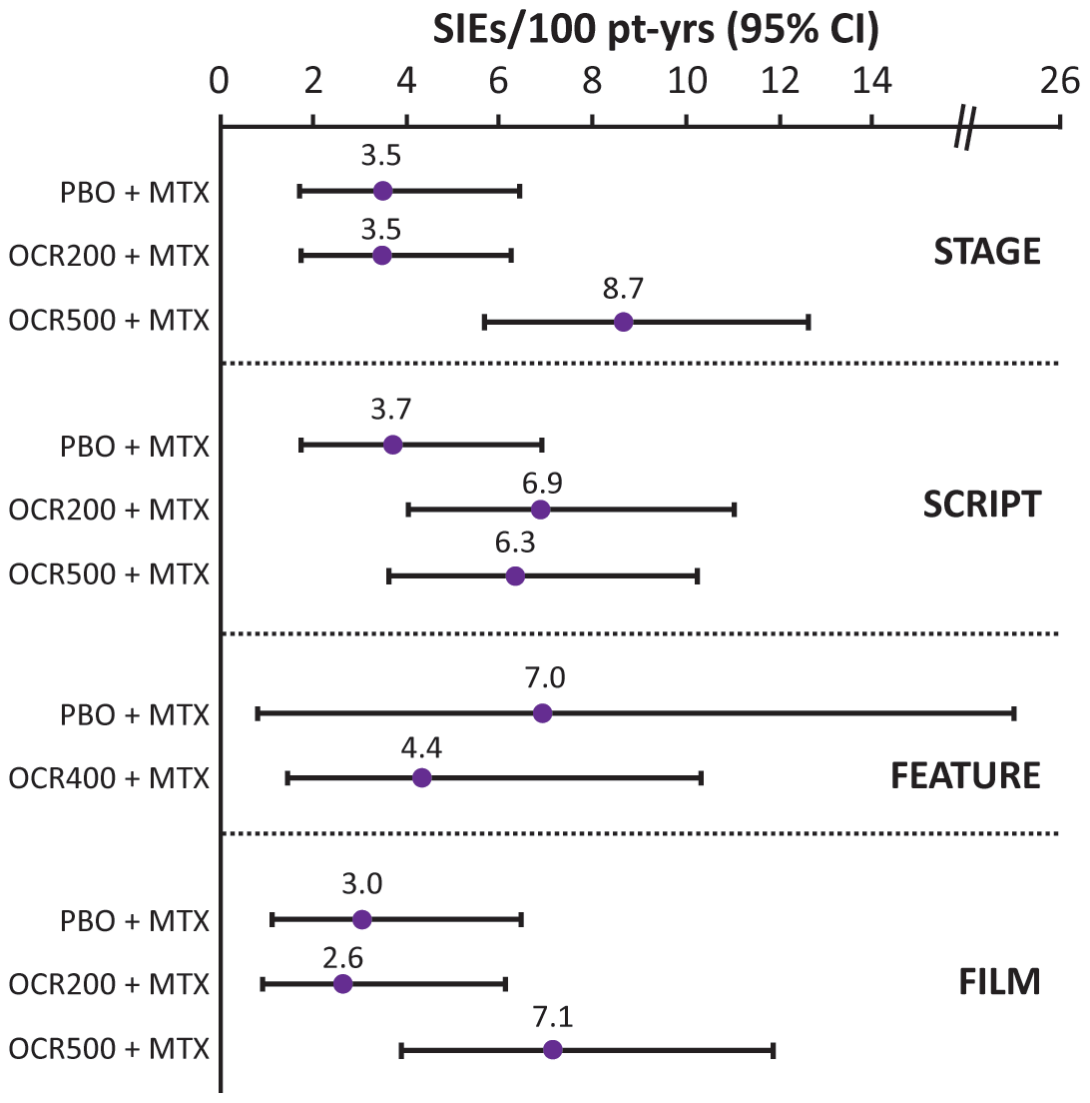
Rates of serious infectious events (SIEs) in the double-blind, placebo-controlled periods. Multiple occurrences of the same event in one individual were counted multiple times. MTX, methotrexate; OCR200, ocrelizumab 200 mg×2; OCR500, ocrelizumab 500 mg×2; PBO, placebo; pt-yrs, patient-years.

In the DBPC periods of FILM, SCRIPT and STAGE, the rates of SIEs (events per 100 patient-years) were higher in the OCR500+MTX group than in the PBO+MTX group (Figure 2). SIE rates were comparable between the OCR200+MTX and PBO+MTX groups in these trials, with the exception of SCRIPT. In SCRIPT, there was also a numerically higher rate of SIEs with OCR200+MTX. The most common types of SIEs in all trials were respiratory tract infections (most frequently pneumonia), cellulitis and urinary tract infections.

**Figure 3:**
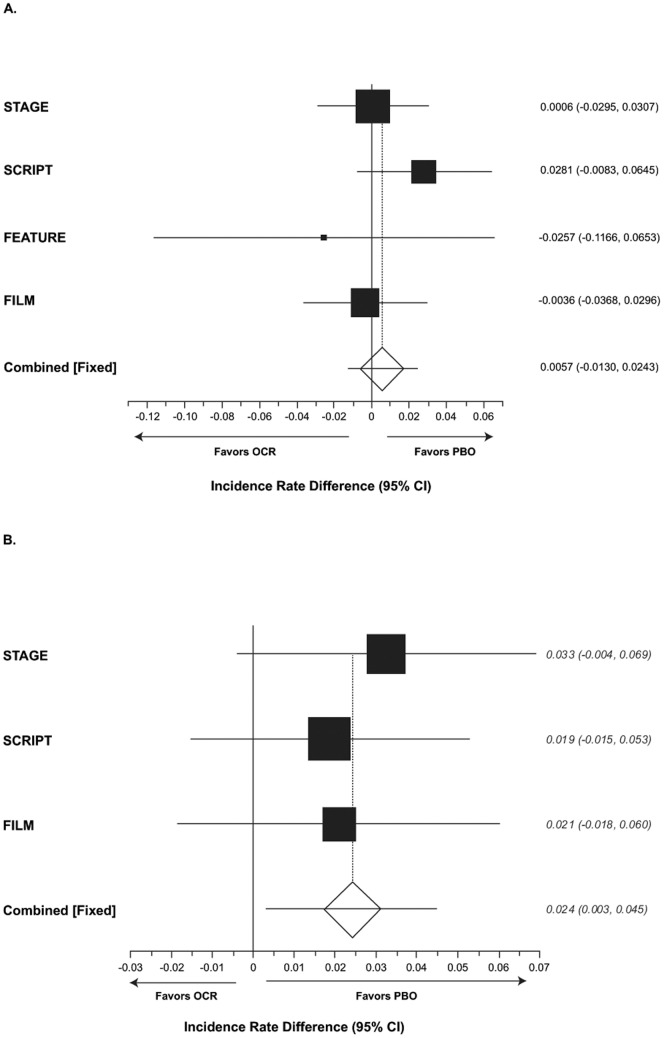
Fixed-effects meta-analysis of incidence rate differences in serious infectious events (SIEs). (A) OCR200+MTX; (B) OCR500+MTX. The pooled rate difference accounts for study and is weighted according to the inverse of the estimated variance. These analyses are based on patients with at least one event (does not count all events). Zero indicates no difference, and a positive value indicates that ocrelizumab (OCR) is worse. MTX, methotrexate; OCR200, ocrelizumab 200 mg×2; OCR500, ocrelizumab 500 mg×2; PBO, placebo.

A meta-analysis of SIEs was conducted. Following pooling of data by treatment group, the weighted difference in incidence rate per 100 patient-years from PBO in patients with SIEs was significantly higher with OCR500+MTX (2.4; 95% CI, 0.3–4.5) but not with OCR200+MTX (0.6; 95% CI, −1.3 to 2.4) (Figure 3, A and B).

**Figure 4:**
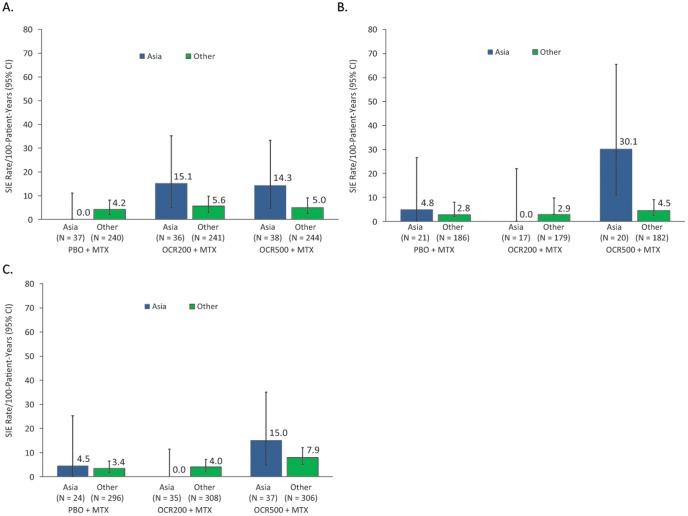
Rates of serious infectious events (SIEs) by region. (A) STAGE; (B) SCRIPT; (C) FILM. MTX, methotrexate; OCR200, ocrelizumab 200 mg×2; OCR500, ocrelizumab 500 mg×2; PBO, placebo. “Asia” includes China, Hong Kong, Indonesia, Malaysia, Philippines, Republic of Korea, Singapore, Taiwan, Thailand and Japan; “Other” includes North and South America, Europe and South Africa.

SIE rates by region for the individual studies (Asia versus Rest of World) showed that SIE rates were particularly high in patients recruited in Asia treated with OCR500+MTX (Figure 4). To explore this further, individual patient data from the larger studies was pooled (STAGE, SCRIPT and FILM) and an exploratory COX regression analysis of risk factors for SIEs was performed. After adjustment for all risk factors in the final model, the following results were found: prior cardiac disease (hazard ratio [HR], 2.29; 95% CI, 1.37–3.83; p = 0.002); use of oral corticosteroids at baseline (HR, 1.69; 95% CI, 1.08–2.65; p = 0.022); history of diabetes (HR, 1.77; 95% CI, 1.02–3.05; p = 0.041); treatment group (relative to PBO+MTX; OCR200+MTX [HR, 1.30; 95% CI, 0.76–2.24; p = 0.341] and OCR500+MTX [HR, 1.87; 95% CI, 1.13–3.11; p = 0.016]); and body weight (≤47.5 kg [5th percentile] relative to >47.5 kg; HR, 2.02; 95% CI, 1.00–4.67; p = 0.049). In addition, after adjustment for these risk factors, patients recruited in Asia appeared to have a higher risk of SIEs compared with those recruited outside of Asia (HR [Asia vs non-Asia] 1.78; 95% CI, 1.03–3.06; p = 0.039). It was not possible to determine whether this effect was driven by race or region because an overwhelming majority of Asian patients were recruited from the Asian region. Study was not significant in the model after adjustment for the risk factors specified. In the exploratory model analyses, there were no statistically significant treatment interactions, including a nonsignificant interaction between Asia and treatment.

Data from the long-term SFU of patients during B-cell depletion post-OCR treatment indicated that the incidence of SIEs decreased over time, and the previously identified imbalance in SIEs in the pivotal studies (STAGE and SCRIPT) in the OCR500+MTX group disappeared. Rates of SIEs in Asian countries also decreased over the long-term follow-up, although it should be noted that the Asian groups consisted of small numbers of patients, and the CIs were wide and overlapped with those of the non-Asian population.

### Opportunistic Infections

A total of 10 opportunistic infections were recorded during the DBPC period. One occurred in the PBO+MTX group (*Mycobacterium abscessus* on the thigh in a patient from Thailand), 5 in the OCR200+MTX group (de novo pulmonary tuberculosis [n = 2; Mexico], hepatitis B reactivation [Japan], *Mycobacterium kansasii* infection [Germany] and histoplasmosis [United States]) and 4 in the OCR500+MTX group (*Pneumocystis jiroveci* [Japan], esophageal candidiasis [France], *Varicella* pneumonia [South Korea] and systemic *Candida* infection [South Korea]). No cases of progressive multifocal leukoencephalopathy were recorded, and no fatal outcomes resulted from opportunistic infection. The patient with hepatitis B virus reactivation tested negative for hepatitis B surface antigen, positive for hepatitis B core antibody and negative for hepatitis B virus DNA at the time of enrollment. Approximately 300 patients with this serologic status for hepatitis B virus were enrolled in the RA program, and no other cases of hepatitis B reactivation were observed.

### Malignancies

Pooled long-term follow-up data (of up to 5 years) from the four studies showed that the rate of malignancies per 100 patient-years was 1.18 (95% CI, 0.61–2.06) in the PBO+MTX group (n = 865; 1018 patient-years), 1.51 (95% CI, 0.99–2.19) in the OCR200+MTX group (n = 1121; 1792 patient-years) and 1.41 (95% CI, 1.07–1.83) in the OCR 500+MTX group (n = 1849; 4034 patient-years). The rate of all active treatments combined (n = 2434; 5826 patient-years) was 1.44 (95% CI, 1.15–1.78). In summary, these pooled analyses revealed no differences in the rate of malignancies between treatment groups.

### Deaths

Overall, 10 deaths occurred during the DBPC periods (Table 3). The 4 deaths in the PBO+MTX groups were due to acute myocardial infarction (n = 2), congestive cardiac failure (n = 1) and rheumatoid vasculitis (n = 1). Of the 6 fatalities among OCR+MTX-treated patients, 4 occurred in patients receiving the OCR500 dose (1 each due to respiratory failure, sepsis, acute myocardial infarction and ischemic stroke) and 2 in patients receiving the OCR200 dose (1 each due to hemorrhagic stroke and acute respiratory failure).

### Pharmacodynamics

**Figure 5:**
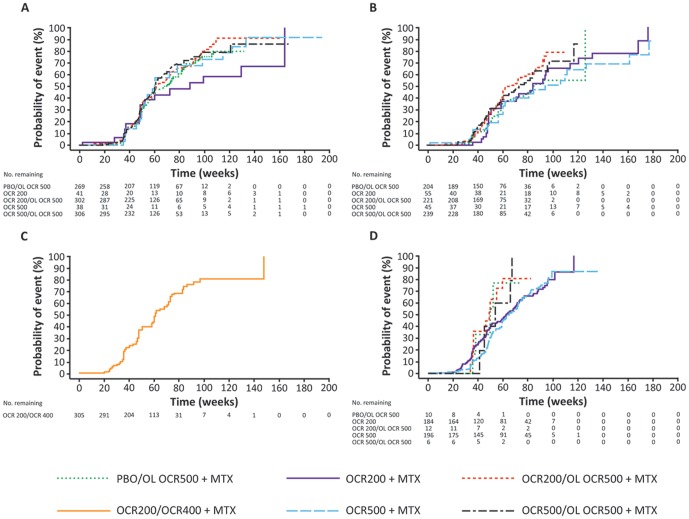
Cumulative probability of B-cell repletion in each clinical trial. (A) STAGE; (B) SCRIPT; (C) FEATURE and (D) FILM. B-cell repletion was defined as a return of CD19+ levels to baseline or 80 cells/µL, whichever was lower. Data summarized under the PBO/OL OCR500+MTX, OCR200/OL OCR500+MTX and OCR500/OL OCR500+MTX treatment groups are OL data only. The x-axis represents the number of weeks since the first infusion of the last course of OCR. MTX, methotrexate; OCR200, ocrelizumab 200 mg×2; OCR500, ocrelizumab 500 mg×2; OL, open-label; PBO, placebo.

In all 4 trials, following the initiation of OCR treatment, a rapid depletion of CD19+ B cells was observed in the OCR200+MTX and OCR500+MTX groups as early as the first post-dose evaluation time point at week 2, in contrast with the PBO+MTX groups. Kaplan-Meier curves of times to B-cell repletion (return of CD19+ levels to baseline or ≥80 cells/µl, whichever was lower) in each of the 4 studies are shown in Figure 5. Data from FILM allowed evaluation of the potential dose effect (after 2–4 courses) on B-cell repletion. No clinically meaningful difference was observed in the median time to B-cell repletion from the first dose of the last course between the OCR200+MTX (63.6 weeks; 95% CI, 53–72) and OCR500+MTX (66.1 weeks; 95% CI, 60–73) groups. In addition, the median times to B-cell repletion were similar between the OCR200+MTX and OCR500+MTX groups in the DBPC period of FILM and between the OCR200/OL OCR500+MTX and OCR500/OL OCR500+MTX groups in the open-label extension of STAGE, respectively, suggesting that a greater number of OCR re-treatments were not associated with a longer repletion time. A slightly more prolonged B-cell repletion profile was observed in SCRIPT when compared with the other studies; this may be related to this patient population having more severe, long-term disease with multiple previous treatments. There was no indication that the time to B-cell repletion in patients recruited in Asia was different from that in patients recruited outside Asia.

## Discussion

This report summarizes the safety results from the 4 phase III trials conducted with OCR in patients with RA. The majority of the population studied included patients with long-standing RA, who had been using numerous immunosuppressive treatments in the past and at least one immunosuppressive agent in combination with OCR during participation in the studies. Approximately one-third of the population studied previously received biological DMARDs and more than one-half of the patients were concomitantly receiving systemic corticosteroids. These factors have to be taken into consideration when interpreting safety data from the OCR clinical trial program in RA.

Although the overall safety profiles were generally comparable between the PBO+MTX and both OCR+MTX dose groups, an imbalance in the incidence of SIEs during the DBPC periods was observed in the OCR500+MTX group. A meta-analysis of SIEs in the DBPC treatment periods indicated a significantly higher rate of SIEs among patients who were treated with OCR500+MTX when compared with PBO+MTX. This was not observed with the lower dose studied. Other factors associated with risk of SIEs were prior cardiac disease, use of oral corticosteroids at baseline, and history of diabetes. Patients recruited in Asia were also at a higher risk of SIEs than were those recruited outside of Asia. Because nearly all Asian patients were recruited in the Asian region, we were unable to distinguish between geographic effects and ethnicity. In addition, the low number of SIEs in the DBPC period meant that we had limited statistical power in the analyses of interactions of risk factors, such as Asian region with treatment.

Confounding factors may have contributed to the higher incidence of opportunistic infections (9 cases across the OCR+MTX groups: 5 cases in the OCR200+MTX group and 4 cases in the OCR500+MTX group compared with a single case in the PBO group) such as endemic areas for histoplasmosis in the United States, tuberculosis in Mexico, and hepatitis B in Japan. In addition, the patient with *Candida* infections was receiving high-dose steroid treatment for concurrent medical conditions.

The clinical development of OCR was initiated in part with the aim of evaluating the potential safety advantage of a humanized molecule over chimeric antibodies. Humanization may be expected to reduce the incidence of anti-drug antibody responses. The incidence of HAHAs was low across the 4 trials (<5%) and, in general, comparable between the pooled OCR+MTX and PBO+MTX groups. There was no association between IRRs and development of HAHAs. In addition, there were no clear differences in the incidence of HAHAs when single-infusion and dual-infusion OCR were compared, although, because the patient numbers in FEATURE were small, the question of whether a difference exists between single- and dual-infusion OCR remains open. In a previous pooled analysis of approximately 2500 patients in the rituximab RA clinical trial program, 11% of those treated with rituximab developed human anti-chimeric antibodies [8].

As expected, both doses of OCR rapidly depleted B cells shortly after infusion. The question was whether the higher rates of serious infections seen in patients treated with OCR500+MTX could have been explained, in part, by differences in B-cell depletion/repletion profiles between the higher and lower doses. It should be noted that evaluation of B-cell levels in clinical trials is limited by measurement of peripheral CD19 counts only; however, the analyses suggested that there was no difference in time to peripheral B-cell repletion between the OCR500 and OCR200 doses. Moreover, the number of repeat treatment courses also did not seem to have a clinically meaningful effect on time to B-cell repletion.

The conclusion that the two doses of OCR, in combination with MTX tested in the RA clinical trials did not demonstrate a superior benefit-risk profile compared with available treatments led to the termination of the clinical development program of OCR in RA. OCR500+MTX demonstrated clinical benefit by improving signs and symptoms of RA and radiographic outcomes [10]–[13]; however this dose was associated with an increased incidence of SIEs. OCR200+MTX did not show superior efficacy compared with existing therapies, but was safe and well-tolerated.

The clinical development of OCR is continuing in multiple sclerosis, for which there remains an unmet need for more effective therapies and background immunosuppressant therapy is not used. A phase II study in multiple sclerosis reported good efficacy and safety data, with no imbalance in serious infections between PBO and OCR (maximum dose up to 1000 mg×2 for 24 weeks) [14]. Phase III studies are continuing and, because of the low prevalence of multiple sclerosis in Asia, no investigational sites in that region have been included.

## Supporting Information

Checklist S1 **CONSORT Checklist.**
(DOC)
